# Comparison of community-onset *Staphylococcus argenteus* and *Staphylococcus aureus* sepsis in Thailand: a prospective multicentre observational study

**DOI:** 10.1016/j.cmi.2016.01.008

**Published:** 2016-05

**Authors:** N. Chantratita, C. Wikraiphat, S. Tandhavanant, G. Wongsuvan, P. Ariyaprasert, P. Suntornsut, J. Thaipadungpanit, N. Teerawattanasook, Y. Jutrakul, N. Srisurat, P. Chaimanee, J. Anukunananchai, S. Phiphitaporn, P. Srisamang, P. Chetchotisakd, T.E. West, S.J. Peacock

**Affiliations:** 1) Department of Microbiology and Immunology, Faculty of Tropical Medicine, Mahidol University, Bangkok, Thailand; 2) Mahidol-Oxford Tropical Medicine Research Unit, Faculty of Tropical Medicine, Mahidol University, Bangkok, Thailand; 3) Department of Clinical Pathology, Sunpasitthiprasong Hospital, Ubon Ratchathani, Thailand; 4) Department of Clinical Pathology, Udon Thani Regional Hospital, Udon Thani, Thailand; 5) Department of Clinical Pathology, Khon Kaen Regional Hospital, Khon Kaen, Thailand; 6) Department of Clinical Pathology, Srinagarind Hospital, Faculty of Medicine, Khon Kaen University, Thailand; 7) Department of Medicine, Khon Kaen Regional Hospital, Khon Kaen, Thailand; 8) Department of Medicine, Udon Thani Regional Hospital, Udon Thani, Thailand; 9) Department of Pediatrics, Sunpasitthiprasong Hospital, Ubon Ratchathani, Thailand; 10) Department of Medicine, Faculty of Medicine, Khon Kaen University, Thailand; 11) Division of Pulmonary and Critical Care Medicine, Department of Medicine, University of Washington, Seattle, WA, USA; 12) International Respiratory and Severe Illness Center, University of Washington, Seattle, WA, USA; 13) London School of Hygiene and Tropical Medicine, London, UK

**Keywords:** Prevalence, sepsis, *Staphylococcus argenteus*, *Staphylococcus aureus*, Thailand

## Abstract

*Staphylococcus argenteus* is a globally distributed cause of human infection, but diagnostic laboratories misidentify this as *Staphylococcus aureus*. We determined whether there is clinical utility in distinguishing between the two. A prospective cohort study of community-onset invasive staphylococcal sepsis was conducted in adults at four hospitals in northeast Thailand between 2010 and 2013. Of 311 patients analysed, 58 (19%) were infected with *S. argenteus* and 253 (81%) with *S. aureus*. Most *S. argenteus* (54/58) were multilocus sequence type 2250. Infection with *S. argenteus* was more common in males, but rates of bacteraemia and drainage procedures were similar in the two groups. *S. argenteus* precipitated significantly less respiratory failure than *S. aureus* (5.2% versus 20.2%, adjusted OR 0.21, 95% CI 0.06–0.74, p 0.015), with a similar but non-significant trend for shock (6.9% versus 12.3%, adjusted OR 0.46, 95% CI 0.15–1.44, p 0.18). This did not translate into a difference in death at 28 days (6.9% versus 8.7%, adjusted OR 0.80, 95% CI 0.24–2.65, p 0.72). *S. argenteus* was more susceptible to antimicrobial drugs compared with *S. aureus*, and contained fewer toxin genes although *pvl* was detected in 16% (9/58). We conclude that clinical differences exist in association with sepsis due to *S. argenteus* versus *S. aureus*.

## Introduction

*Staphylococcus argenteus* is a genetically divergent lineage of *Staphylococcus aureus* that received formal taxonomic classification in 2014 [1]. *S. argenteus* is mis-identified as *S. aureus* in clinical diagnostic laboratories using standard identification tests [2], [3], [4]. Multilocus sequence typing (MLST) makes a clear distinction between the two species, but the original PCR primers for *S. aureus* failed to amplify two of the seven loci in *S. argenteus* [2], [5], [6] and such isolates were often excluded by default. Although several of the earliest descriptions of *S. argenteus* were associated with remote communities [2], [3], [4], cumulative publications have since suggested that *S. argenteus* is globally distributed. This includes its isolation from humans or animals in Thailand, Cambodia, Indonesia, Fiji, New Zealand, Mayotte, Trinidad and Tobago, with additional evidence from the MLST database of its presence in Europe and Africa [2], [3], [4], [5], [6], [7], [8], [9], [10], [11], [12].

Discovering the existence of this new pathogenic species results in the need to determine whether *S. argenteus* and *S. aureus* should be distinguished apart in routine practice. This might be necessary if *S. argenteus* infection is associated with different clinical manifestations, severity or outcome, and/or requires different antimicrobial regimens. Data on clinical features of human *S. argenteus* infection are limited, but a study that defined the frequency of *S. argenteus* in three different clinical collections in northern Australia reported that this was predominantly associated with skin and soft-tissue infections, but rarely with bacteraemia [13]. *S. argenteus* has been reported to be more susceptible to oxidative stress and neutrophil killing *in vitro*, and less virulent in murine sepsis and skin infection models compared with *S. aureus* [13]. This raises the possibility that human *S. argenteus* infection may be associated with a milder course. Comparison between ten patients with invasive *S. argenteus* infection and 236 patients with invasive *S. aureus* infection reported that morbidity and death were comparable [6], but this study was underpowered. The presence of virulence genes in *S. argenteus* has not been subjected to detailed investigation. Several studies have reported that *S. argenteus* is negative for genes encoding Panton–Valentine leukocidin (PVL) [3], [4], [7], [8], [14], [15], although a recent case report of two patients with PVL-positive *S. argenteus* infection [11] indicates that this species may acquire virulence factors. One study also detected the gene encoding staphylococcal enterotoxin B in all *S. argenteus* isolates cultured from villagers in the Amazonian forest [4].

Here, we describe a prospective, multicentre study of invasive staphylococcal infection that was initially reported by diagnostic laboratories to be due to *S. aureus*, in which around one-fifth of isolates were re-identified as *S. argenteus*. This allowed a comparison of patient characteristics and demography, features of infection and outcome, antimicrobial resistance profiles, and the presence of selected putative virulence determinants.

## Methods

### Study setting and design

A prospective cohort observational study of community-onset invasive staphylococcal infection was conducted in patients with sepsis at four hospitals across northeast Thailand between March 2010 and December 2013. Study sites were Sunpasitthiprasong Hospital, Ubon Ratchathani; Udon Thani Hospital, Udon Thani; Srinagarind Hospital, Khon Kaen; and Khon Kaen Hospital, Khon Kaen. Potential study patients were identified by daily screening at each hospital diagnostic microbiology laboratory for clinical samples that grew a pure culture identified by the routine microbiology laboratory as *S. aureus*. Invasive infection was defined as the isolation of *S. aureus* from a sample taken from a normally sterile site. Inclusion criteria were as follows: age at least 14 years (age of admission to adult wards), community-onset infection (positive culture taken within 2 days of hospital admission, or after 2 days when sampling was delayed from a patient admitted with suspected infection), and at least two of four systemic inflammatory response syndrome criteria met within 48 h of culture. These criteria are: (a) temperature >38°C or <36°, (b) heart rate >90 beats/min, (c) respiratory rate >20 breaths/min, Pco_2_ <32 mmHg, or a requirement for mechanical ventilation, and (d) white blood count >12 000 cells/mL or <4000 cells/mL, or >10% band forms [16]. Exclusion criteria were other active co-infections, therapeutic immunosuppression with high-dose steroids or chemotherapy, and pregnancy. Patients were enrolled after providing written informed consent.

Clinical information was obtained from the medical records. Patients were followed until hospital discharge, during which time antimicrobial therapy, surgical drainage procedures, the development of shock requiring vasopressors or inotropes, or respiratory failure requiring mechanical ventilation were recorded. Mortality was ascertained 28 days from the day of admission. For patients discharged before 28 days, outcome was determined through telephone follow up. Patient residence was defined using http://mondeca.com/index.php/en/?option=com_content&view=article&id=206&Itemid=752, and the coordinates were mapped using ArcGIS software (https://www.arcgis.com). Ethical approval was obtained from the following Ethical and Scientific Review committees: Faculty of Tropical Medicine, Mahidol University (approval no. MUTM 2011-007-01); Sunpasitthiprasong Hospital, Ubon Ratchathani (approval no. 004/2553); Udon Thani Hospital, Udon Thani (approval no. 0027.102/2349); Khon Kaen Hospital, Khon Kaen; and Faculty of Medicine (Srinagarind Hospital), Khon Kaen University, Khon Kaen, Thailand (approval no. HE541113).

### Culture and antibiotic susceptibility testing

Following initial isolation and identification by the routine diagnostic laboratories, the staphylococcal isolate that triggered enrolment for each patient was stored in trypticase soy broth with 15% glycerol at –80°C before further characterization. Twelve antimicrobial drugs were selected for susceptibility testing based on their prescription at least once for the study cohort. These were cefoxitin, clindamycin, trimethoprim-sulfamethoxazole, erythromycin, fosfomycin, fusidic acid, gentamicin, levofloxacin, oxacillin, penicillin, tigecycline and vancomycin. Antimicrobial susceptibility profiles were determined and interpreted according to the CLSI guidelines (M100-S24) [17], with the exception of fusidic acid and tigecycline, which were tested using the EUCAST guidelines (Version 4.0, 2014; http://www.eucast.org). Zones of inhibition were interpreted using M100-24, and isolates were reported as susceptible, intermediate, or resistant. Isolates were initially assigned as MRSA based on the cefoxitin disk diffusion assay [17], and confirmed using PCR to detect the *mecA* gene.

### *Staphylococcus aureus* genotyping and detection of putative virulence genes

Pulsed-field gel electrophoresis (PFGE) was performed using the *Sma*I restriction enzyme (New England Biolabs, UK) [18]. Banding patterns were analysed using BioNumerics software version 6.01 (Applied Maths, Sint-Martens-Latem, Belgium) as described [19]. A similarity coefficient of 80% was used to define PFGE types [18]. DNA was extracted and MLST was performed as described elsewhere [2], [6], [20]. Sequence types (ST) were assigned using the MLST database (www.mlst.net) [20]. Phylogenetic analysis of concatenated MSLT data was performed using ClustalW for the alignment and the maximum likelihood method for construction of the phylogenetic tree in MEGA version 6. Two multiplex PCR assays were used to detect the presence of virulence and other genes (assay 1 for *tst*, *eta, etb*, *sea*, *seb*, *sec*, *sed*, *see* [21], assay 2 for *mecA* and *pvl*) [22].

### Statistical analysis

Continuous variables are reported as mean and standard deviation for normally distributed data or median and interquartile range for non-normally distributed data. Univariable analyses were performed with logistic regression. Multivariable analyses were performed with multiple logistic regression, adjusting as appropriate for age, sex, co-morbidities, centre, delayed initiation of anti-staphylococcal antibiotics and surgical drainage of infection. A co-morbidity index (possible range one to ten) assigned one point for each of the following pre-existing medical conditions: lung disease, heart disease, kidney disease, liver disease, neurological disease, haematological disease, autoimmune disease, cancer, diabetes and alcoholism. Statistical analysis was conducted using Stata v11.2 (College Station, TX, USA).

## Results

### Study patients

A total of 327 patients with sepsis meeting the inclusion criteria were enrolled. The bacterial isolate was discarded in error for 16 patients and these cases were excluded from the analysis. Recruitment was highest at Sunpasitthiprasong Hospital (*n* = 154), followed by Udon Thani Hospital, Khon Kaen Hospital, and Srinagarind Hospital (*n* = 99, *n* = 34 and *n* = 24, respectively). Characteristics of the 311 study patients at the time of enrolment are shown in Table 1. The median age was 53 years (interquartile range 41–65), and 108 (34.7%) were women. Nearly half of cases (*n* = 149, 47.9%) had no pre-existing medical condition, but a third (*n* = 109, 35.1%) had diabetes mellitus. In all, 119 patients (38.3%) had documented staphylococcal bacteraemia at the time of enrolment. Several patients had positive cultures from multiple sites, in which case the first isolate was obtained for analysis. The types of specimens from which isolates included here were cultured were abscesses (*n* = 163, 52.4%), blood (*n* = 115, 37.0%), bone or arthocentesis samples (*n* = 21, 6.8%), body fluids including peritoneal, pleural, biliary tract and cerbrospinal fluid samples (*n* = 7, 2.3%), pus from implanted surgical hardware (*n* = 3, 1.0%), and pus from spaces such as sinuses and inner ear (*n* = 2, 0.6%). Anti-staphylococcal therapy was initiated no more than 2 days after cultures were taken in 304 patients (97.7%). A total of 196 patients (63.0%) underwent a procedure to drain pus. Sixty-six patients (21.2%) developed respiratory failure or shock. All-cause mortality was 9% at 28 days, with a higher death rate in patients with documented bacteraemia at the time of enrolment (19.3%) or in those who developed respiratory failure or septic shock (33.3%).

### Prevalence of *S. argenteus* infection

**Fig. 1:**
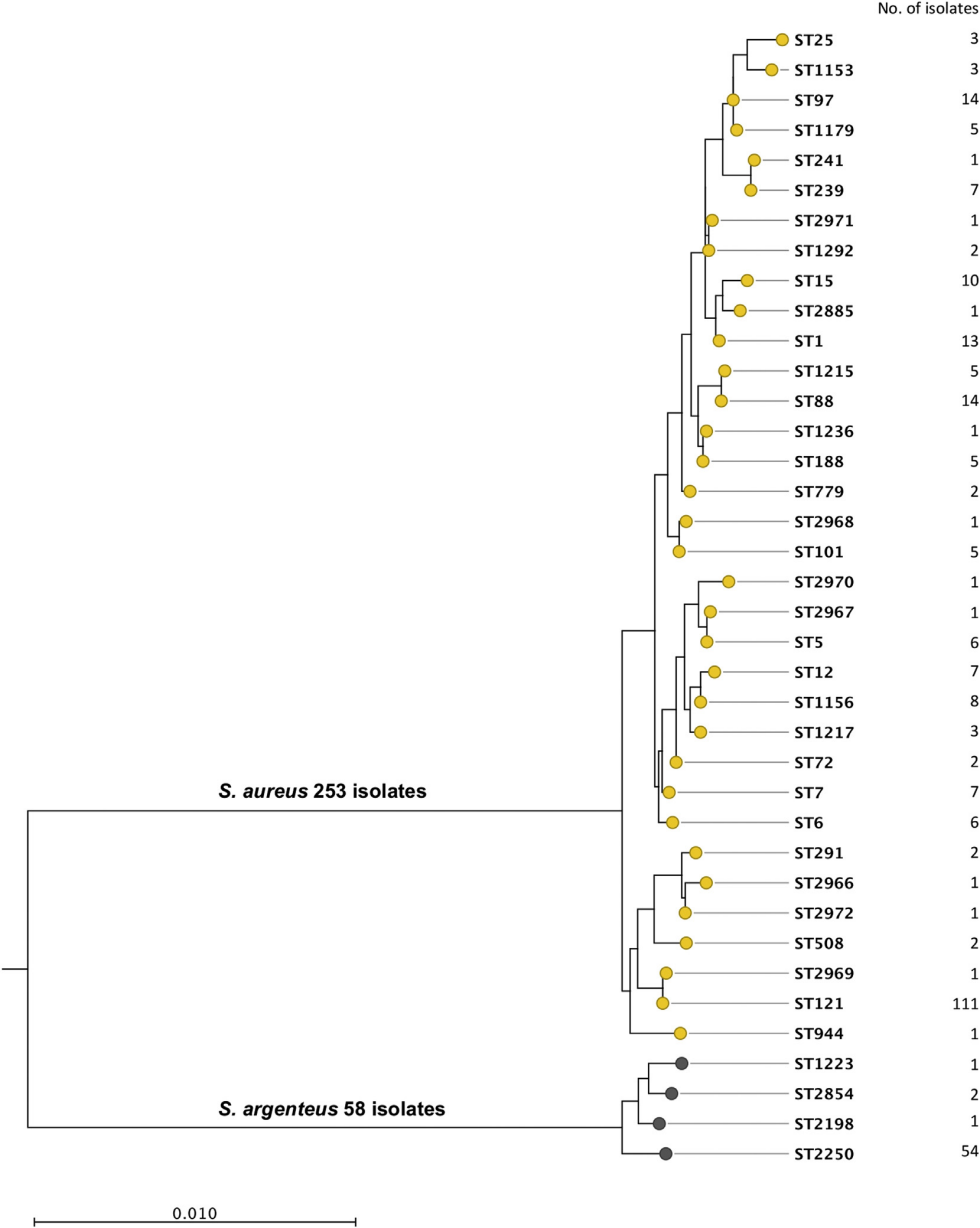
Phylogenetic tree of 311 putative *Staphylococcus aureus* isolates. The tree is based on concatenated sequences of seven multilocus sequence typing loci using the maximum likelihood method.

**Fig. 2:**
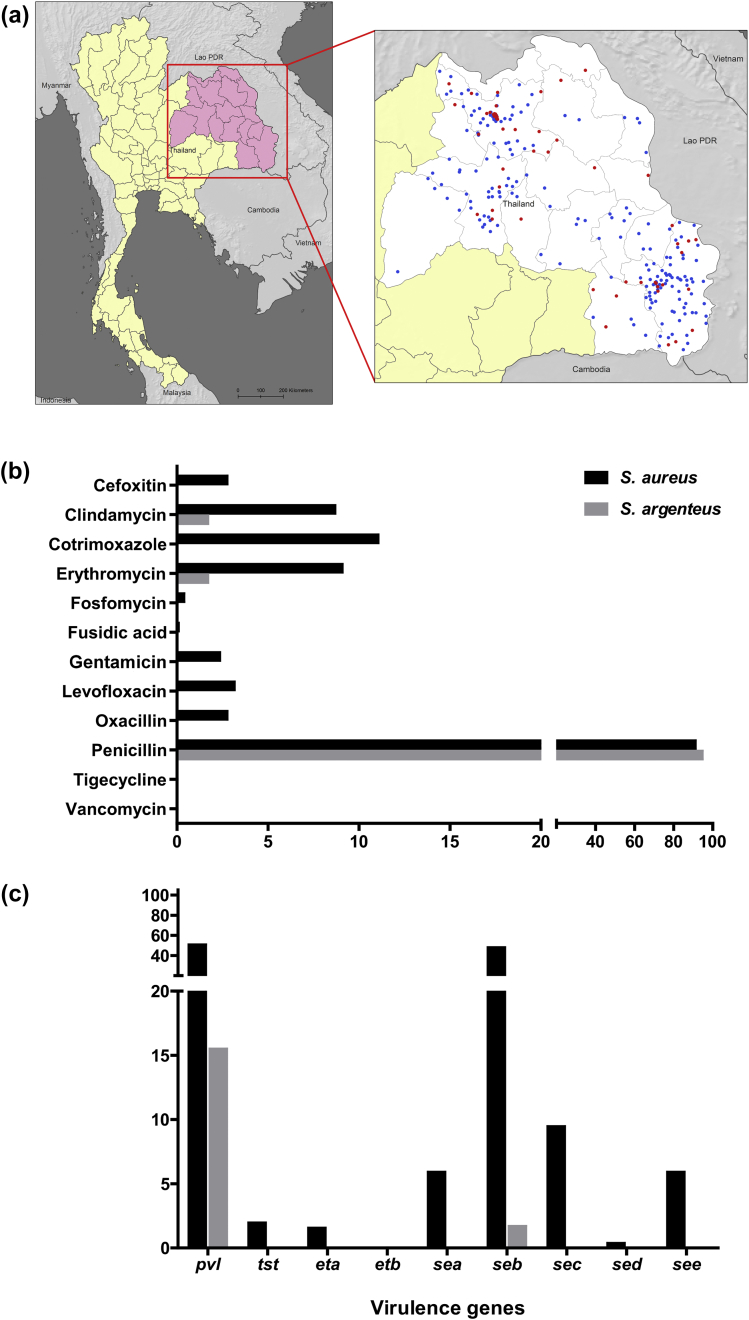
Comparisons between *Staphylococcus argenteus* and *Staphylococcus aureus*. (a) Geographic distribution of patient residence before hospital admission. Blue and red dots represent patients infected with *S. aureus* (*n* = 253) and *S. argenteus* (*n* = 58), respectively. (b) Antimicrobial resistance and (c) presence of selected putative virulence genes in *S. aureus* (*n* = 253) and *S. argenteus* (*n* = 58) isolates, in per cent.

A combination of PFGE and MLST was used to distinguish between *S. argenteus* and *S. aureus*. PFGE was performed on all 311 isolates and MLST was then undertaken for at least one representative of each of the PFGE banding pattern types. PFGE revealed 71 different banding patterns, MLST of which resolved 38 STs. The most common STs were ST121 (*n* = 111, 36%) and ST2250 (*n* = 54, 17%), while ST88 and ST97 each comprised 14 (5%) isolates and ST1 comprised 13 (4%) isolates. The remaining 105 isolates belonged to 33 different STs, seven of which were novel (ST2966, ST2967, ST2968, ST2969, ST2970, ST2971 and ST2972). Six of the seven MRSA isolates were ST239 (the prevalent MRSA clone in Thailand) and the remaining isolate was a single locus variant of ST239 (ST241). A maximum likelihood phylogenetic tree based on concatenated sequences of seven MLST loci divided the collection into two major branches containing 253 (81%) and 58 (19%) isolates, respectively (Fig. 1). The branch containing 253 isolates was defined as *S. aureus* based on assignment to, or clustering with previously established *S. aureus* lineages. The 58 isolates in the second cluster were all defined as *S. argenteus* based on belonging to STs that have been assigned previously to this species. The *S. argenteus* branch contained four STs, but the overwhelming majority (54/58, 93%) belonged to ST2250. *S. argenteus* was isolated with varying frequency depending on the hospital; it accounted for 30% (95% CI 21%–40%) of isolates from Udon Thani Hospital, 25% (95% CI 10%–47%) from Srinagarind Hospital, 13% (95% CI 8%–19%) from Sunpasitthiprasong Hospital, and 6% (95% CI 1%–20%) from Khon Kaen Hospital. Mapping the place of residence for patients infected by each of the two staphylococcal species demonstrated that patients infected with *S. argenteus* and *S. aureus* were drawn from a comparably broad range of locations across northeast Thailand (Fig. 2a). All *S. argenteus* isolates had a white colony morphology consistent with the lack of staphyloxanthin.

### Clinical comparison between *S. aureus* and *S. argenteus* sepsis

To determine whether S*. argenteus* causes sepsis in different populations than those infected with *S. aureus*, we compared patient characteristics between the 253 patients with *S. aureus* infection and 58 patients with *S. argenteus* infection (Table 1). There was no difference in age between the two groups but *S. argenteus* infection was less common in females (adjusted OR 0.45, 95% CI 0.22–0.90, p 0.03). The odds ratio point estimates suggested that heart disease and cancer were more common in patients with *S. argenteus* infection, although the confidence intervals were wide (adjusted OR 2.19, 95% CI 0.67–7.21, p 0.20, and adjusted OR 2.64, 95% CI 0.44–16.00, p 0.29). To evaluate the previous suggestion that *S. argenteus* may be less virulent than *S. aureus*, we compared rates of bacteraemia, treatments and clinical outcomes between the 58 patients with *S. argenteus* infection and 253 patients with *S. aureus* infection. Bacteraemia was comparably likely in patients with *S. argenteus* infection (32.8% versus 39.5%, adjusted OR 0.65, 95% CI 0.34–1.24, p 0.19). There was no difference in rates of delayed antibiotic administration (2.8% versus 0.0%, p 0.36) or in rates of drainage procedures performed in both groups (62.1% vs 63.2%, adjusted OR 1.21, 95% CI 0.63–2.30, p 0.57). Clinical outcomes are shown in Table 2. *S. argenteus* infection precipitated less respiratory failure than *S. aureus* infection during hospitalization (5.2% versus 20.2%, adjusted OR 0.21, 95% CI 0.06–0.74, p 0.015) and the odds ratio point estimate for shock trended in the same direction (6.9% versus 12.3%, adjusted OR 0.46, 95% CI 0.15–1.44, p 0.18). There was no difference in the rates of death at 28 days (6.9% versus 8.7%, adjusted OR 0.80, 95% CI 0.24–2.65, p 0.72) for *S. argenteus* and *S. aureus*, respectively.

### Bacteriological comparisons between *S. argenteus* and *S. aureus*

A bacteriological comparison was undertaken between the two staphylococcal species to determine rates of antimicrobial drug resistance, and the presence of toxin genes found in *S. aureus*. The antimicrobial drugs tested were those prescribed at least once to the study cohort. Rates of resistance to penicillin were similar between the two species, but rates of resistance were notably lower in *S. argenteus* for the remainder of the drugs tested (Fig. 2b). Specifically, *S. argenteus* resistance was only observed in a single isolate for two additional drugs (erythromycin and clindamycin). PCR was used to define the presence of nine toxin genes in the two species (Fig. 2c). Only two toxin genes (*pvl* and *seb*) were detected in *S. argenteus*, which were present at a frequency of 16% (9/58) and 2% (1/58), respectively. All nine *pvl*-positive isolates were confirmed by MLST as ST2250. By comparison, eight of the genes tested were detected in *S. aureus* at a variable frequency (Fig. 2c). *pvl* was detected in 51% (129/253) of *S. aureus*, which is consistent with previous studies based in the same geographical region [22].

## Discussion

This study indicates that *S. argenteus* is an important cause of community-acquired invasive methicillin-susceptible *S. aureus* sepsis in Thailand. Our data suggest an increase in the prevalence of invasive, community-acquired *S. argenteus* infection in northeast Thailand since 2006/07, when 4% of isolates from 246 unselected patients presenting to Sunpasitthiprasong Hospital (one of the study hospitals) with invasive putative *S. aureus* disease were actually *S. argenteus* [6]. The basis for this is unclear, although we note that *S. argenteus* sepsis occurred more often in men and may be associated with occupation or differential exposure for other reasons.

Apart from the dominant *S. argenteus* ST2250, three other STs—ST2198, ST1223 and ST2854—were also identified for this species. These STs have been reported previously from Cambodia, Australia and French Guiana (ST1223); the UK (ST2250); Australia and Germany (ST2198); and Thailand (ST2854) [2], [4], [5], [6], [14]. *S. argenteus* was previously reported as the cause of community infections in South East Asia [2], Australia [3], South America [4] and Europe (http://saureus.mlst.net/). The most common ST among *S. aureus* isolates was ST121, a finding that is concordant with previous reports demonstrating that ST121 is common in Thailand [6], Cambodia [23] and other countries [24], [25], [26].

A key objective of this study was to understand whether there was utility in determining whether a patient with invasive staphylococcal disease was infected by *S. aureus* or *S. argenteus*. This is unlikely to influence the choice of antimicrobial treatment since susceptibility testing will dictate prescribing, but could be important if disease severity was clearly different between the two. In comparison with individuals with *S. aureus* infection, patients with *S. argenteus* infection may be more likely to have heart disease or cancer, although we could not establish this conclusively due to wide confidence intervals around the odds ratio point estimates. Bacteraemia is often associated with more severe disease manifestations, but rates of bacteraemia were comparable between the two groups. There was no difference in 28-day mortality, yet there was a notably lower rate of respiratory failure in patients with *S. argenteus* infection. A similar trend was observed for septic shock, although this did not reach statistical significance. Importantly, this observational study was underpowered to detect differences in rates of less common outcomes such as septic shock and death.

There are several potential explanations for the reduced respiratory failure in patients with *S. argenteus* sepsis. This species is more susceptible to antimicrobial drugs overall, and may be less likely to be treated with an empiric agent to which it is resistant. Although biochemical phenotypes and 16S rRNA sequence are similar to *S. aureus*, there is approximately 10% core genome divergence from prototypical *S. aureus* [14]. A range of virulence determinants found in *S. aureus* may be absent in *S. argenteus. S. argenteus* lacks staphyloxanthin, a carotenoid pigment that confers resistance against oxidative stress and neutrophil killing [27], [28]. This observation has led others to hypothesize that *S. argenteus* may be less virulent than *S. aureus* [14]. A single test isolate of *S. argenteus* was more susceptible to oxidative stress and neutrophil killing *in vitro* and less virulent in murine sepsis and skin infection models compared with *S. aureus* [27], [28], but this was not fully explained by the action of staphyloxanthin because the same isolate expressing the gene encoding staphyloxanthin was more resistant to oxidative stress *in vitro* but showed no change in resistance to neutrophil killing or *in vivo* virulence [13]. The majority of *S. argenteus* were negative for the genes encoding PVL, although the finding that this was present in a proportion of *S. argenteus* isolates represents the second description of *pvl*-positive *S. argenteus* to our knowledge after a report of two cases that presented in 2014 [11].

Our results should be interpreted in light of several limitations. We did not enrol patients who died rapidly following admission, reducing our power to detect associations with death. Moreover, the total number of patients enrolled was relatively small, often resulting in wide confidence intervals around point estimates. Hence, although we have identified several important clinical differences in patients with *S. argenteus* infection compared with those with *S. aureus* infection, we cannot exclude other clinically relevant associations that our study lacked the power to identify. Furthermore, most *S. argenteus* isolates belonged to a single ST, and further studies are required to compare the two diseases in other settings where the prevalent lineages of this species differ. Finally, despite our efforts to control for potential confounding factors, residual confounding may still exist. However, strengths of our study are prospective patient enrolment at multiple centres in northeast Thailand, rigorous enrolment criteria requiring clinical sepsis attributable to staphylococcal infection, and more extensive batched analysis of isolates in a central location.

In conclusion, clinical differences exist in patients who develop infection with *S. argenteus* versus *S. aureus* sepsis. *S. argenteus* precipitated significantly less respiratory failure than *S. aureus*, with a similar but non-significant trend for shock. This may be related to heightened antimicrobial susceptibility of *S. argenteus* and expression of fewer toxin genes.

## Funding

SJP was supported by grants from the UKCRC Translational Infection Research (TIR) Initiative, and the Medical Research Council (Grant Number G1000803) with contributions to the Grant from the Biotechnology and Biological Sciences Research Council, the National Institute for Health Research on behalf of the Department of Health, and the Chief Scientist Office of the Scottish Government Health Directorate. NC was supported by a Wellcome Trust Career Development award in Public Health and Tropical Medicine (087769/Z/08/Z).

## Transparency declaration

All authors declare that they have no conflicts of interest.

## Authors' contributions

NC, TEW and SJP were involved in the design of the study. CW, PA, PSu, PSr, SP, JA and PChe recruited patients and collected metadata. GW, NT, YJ, NS and PCha provided bacterial isolates, and undertook antimicrobial susceptibility testing. CW, ST, JT and NC performed the genotyping and genotype analysis. TEW performed the statistical analysis. NC was responsible for supervision of the study. All authors were involved in writing the paper and have approved the final version.

## Figures and Tables

**Table 1:** Characteristics of patients with *Staphylococcus aureus* or *Staphylococcus argenteus* infections

Variable	All cases (*n* = 311)	*S. aureus* (*n* = 253)	*S. argenteus* (*n* = 58)	Crude OR (95% CI) p value	Adjusted OR (95% CI) p value
Median age, years (IQR)	53 (41–65)	53 (40–65)	56.5 (47–67)	1.01 (0.99–1.03) 0.25	1.01 (0.99–1.03) 0.55
Female, *n* (%)	108 (34.7)	93 (36.8)	15 (25.9)	0.60 (0.32–1.14) 0.12	0.45 (0.22–0.90) 0.03
Pre-existing conditions, *n* (%)					

Diabetes mellitus	109 (35.1)	84 (33.2)	25 (43.1)	1.52 (0.85–2.73) 0.16	1.57 (0.81–3.05) 0.19
Heart disease	16 (5.1)	10 (4.0)	6 (10.3)	2.80 (0.98–8.06) 0.09	2.19 (0.67–7.21) 0.20
Kidney disease	51 (16.4)	40 (15.8)	11 (19.0)	1.25 (0.60–2.61) 0.56	0.93 (0.38–2.25) 0.87
Liver disease	11 (3.5)	10 (4.0)	1 (1.7)	0.43 (0.05–3.40) 0.42	0.29 (0.03–2.65) 0.28
Lung disease	17 (5.5)	15 (5.9)	2 (3.5)	0.57 (0.13–2.55) 0.46	0.42 (0.08–2.07) 0.29
Cancer	7 (2.3)	5 (2.0)	2 (3.5)	1.77 (0.34–9.37) 0.50	2.64 (0.44–16.00) 0.29
Alcoholism	4 (1.3)	4 (1.6)	0	—	—

Autoimmune disease	5 (1.6)	4 (1.6)	1 (1.7)	1.09 (0.12–9.96) 0.94	0.66 (0.06–7.16) 0.74
Neurological disease	8 (2.6)	7 (2.8)	1 (1.7)	0.62 (0.07–5.11) 0.65	0.63 (0.07–5.79) 0.69
Haematological disease	8 (2.6)	5 (2.0)	3 (5.2)	2.71 (0.63–11.66) 0.18	1.67 (0.32–8.72) 0.55
Median co-morbidity index (IQR)	1 (0–1)	1 (0–1)	1 (0–1)	1.23 (0.91–1.68) 0.19	1.16 (0.82–1.63) 0.39

IQR, interquartile range.

**Table 2:** Outcomes of patients with *Staphlococcus aureus* or *Staphylococcus argenteus* infections

Pathogen	Outcome		Crude OR (95% CI) p value	Adjusted OR (95% CI) p value
No	Yes
**Respiratory failure**				
*S. aureus*	202 (79.8)	51 (20.2)	0.22 (0.06–0.72) 0.012	0.21 (0.06–0.74) 0.015
*S. argenteus*	55 (94.8)	3 (5.2)
**Shock**				
*S. aureus*	222 (87.8)	31 (12.3)	0.53 (0.18–1.57) 0.25	0.46 (0.15–1.44) 0.18
*S. argenteus*	54 (93.1)	4 (6.9)
**Death**				
*S. aureus*	231 (91.3)	22 (8.7)	0.78 (0.26–2.36) 0.66	0.80 (0.24–2.65) 0.72
*S. argenteus*	54 (93.1)	4 (6.9)
